# Characterization of the Volatile Substances and Aroma Components from Traditional Soypaste

**DOI:** 10.3390/molecules15053421

**Published:** 2010-05-11

**Authors:** Yan Zhang, Xin Li, Chih-Kang Lo, Shun-Tang Guo

**Affiliations:** 1 Central Research Institute of Ting Hsin International Group, Tianjin 300457, China; E-Mails: healscience@126.com (Y.Z.); patrick@tinghsin.com.cn (C.-K.L.); 2 College of Food Science and Nutritional Engineering, China Agricultural University, Beijing 100083, China; E-Mail: lixin250007@126.com (X.L.)

**Keywords:** flavor, soypaste, aroma, GC-MS, GC-O, FD value, AEDA

## Abstract

In this study, the flavor substances of soypaste were extracted by a simultaneous distillation method and identified by GC-MS. The characteristic aroma components of soypaste were determined by the GC-O technique and the FD value of the characteristic aroma components was determined by AEDA method. It could be inferred that the aroma of the soypaste should be attributed to the presence of heterocyclic compounds and organic acids, with the heterocyclic compounds playing a prominent role.

## 1. Introduction

Soypaste is a kind of special seasoning produced by microbial fermentation using soybean and wheat flour as the main raw materials. Traditional soypaste has a unique aroma due to the natural fermentation production method. However, the production of the soypaste suffers from a long production cycle, high costs and low output. Many soypaste manufacturers use artificial fermentation installations and other industrial fermentation processes to expand the scale of production and reduce the cost, but significant differences in aroma are noted by the consumers, which has also become a major factor in markg acceptance. Thus, the aroma of soypaste is considered as one of the important indicators to evaluate the quality of soypaste.

Until recently, soypaste aroma research of was still in the development. The research was focused on the identification and the extraction methods of aroma components [1,2,3,4] and the results only showed the aroma substances in the samples by similar methods such as solid-phase micro-extraction and the direct distillation method, which could not further specify which were the main flavor ingredients. As a result, it was difficult to establish the industrial standard from the aspect of the characristic aroma and the functions of different favor components in traditional soypaste still could not be determined. In this study, the flavor substances of soypaste were extracted by simultaneous distillation method and identified by GC-MS. Then the characteristic aroma components in the soypaste were determined by the GC-O analysis technique and the FD values of the characteristic aroma components were measured by the AEDA method. The results obtained could be used to understand the aroma of traditional soypaste and help standardize the modern product of soypaste.

## 2. Results and Discussion 

**Figure 1:**
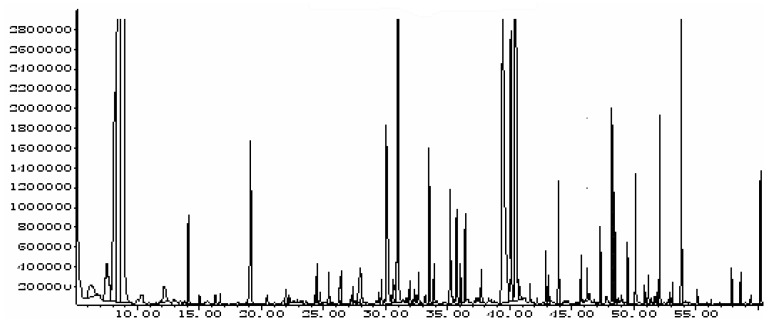
GC profile of the volatile components extracted from the soypaste by SDE.

The GC profile of the volatile components extracted from the soypaste by SDE (simultaneous distillation and extraction) is shown in Figure 1. One hundred and eight eight components were detected in soypaste extracts, among which 103 compounds were identified by searching the MSDChem NIST02.1 MS library (similarity ratio >75%). Through the data processing system of the MSDChem ChemStation, the relative contents of the various components in the volatile oil were determined by the peak area normalization method and the identified compounds thus accounted for over 80% of the overall aroma components. 

In accordance with the order of the peaks, the main identified compounds were as follows: 2-methyl propionaldehyde (0.6568%), ethyl acetate (0.9856%), 3-butyraldehyde (4.4674%), ethanol (16.0962%), 2-methyl-1-propanol (0.5181%), formic acid ester (0.1137%), heptanal (0.0137), *iso*-amyl alcohol (1.5799%), 2-pentylfuran (0.1063%), 2,5-dimethylpyrazine (0.2044%), 2,6-dimethylpyrazine (0.1038%), ethyl lactate (0.2514%), dimethyl trisulfide (0.1145%), 3-methylpyrazine (0.1862%), acetic acid (2.1983%), 3-methylthiopropionaldehyde (0.168%), furfural (7.5069%), 2-acetylfuran (0.2062%), benzaldehyde (0.9816%), 2-methylpropionic acid (0.9689%), 5-methylfurfural (0.7121%), phenyl-acetaldehyde (6.5578%), furfuryl alcohol (2.1997%), 3-methyl-pentanoic acid (7.0269%), ethyl phenylacetate (0.188%), guaiacol (0.6268 %), phenethyl alcohol (0.7545%), 2-acetylpyrrole (0.9778%), 4-ethylguaiacol (3.2858%), 4-ethylphenol (0.2072%) and palmitic acid ethyl ester (0.8126%).

In this study, 103 volatile compounds were identified in traditional soypaste, mainly esters, alcohols, aldehydes, acids, ketones, and heterocyclic compounds. The contents of alcohols and acids were the highest, followed by the contents of esters and aldehydes. Although the contents of alcohols were high, they had a relatively high threshold, which made their contribution to the flavor small. Esters were the main component present in the soypaste aroma volatile components, providing sweet bean paste, fruit fragrance, and a variety of floral aromas. The aldehydes and ketones had the lowest thresholds and produced fruit fragrances and nut aromas. The heterocyclic compounds were the main component of the cconstituted the main body of the baking aroma. High contents of some compounds such as ethyl acetate, benzoin aldehyde, 5-methylfurfural, benzene, acetaldehyde, furfural, 3-butyric aldehyde, 2,3,5-trimethylpyrazine, *etc*., some of which had been identified as flavor compounds, have been detected in the soypaste.

The characteristic aroma components of the soypaste were determined by the GC-O technique. During the GC-O analysis, there were three professional evaluators to detect 22 kinds of odor active volatile compounds (Log3 FD> 1). These compounds were classified according to the sequence in Table 1, Table 2, Table 3, Table 4 and Table 5. Many different odors were sniffed at the GC-O sniffing port, including coke flavor, fried potato flavor, fruit fragrance, flower fragrance, butter flavor and so on. All the compounds could be detected in GC-MS. The Log3 FD values of 18 compounds were more than 3. Of these 2,3,5-trimethyl-pyrazine (a strong aroma of fried potatoes), isovaleric (smelly sock smell), 4-ethylguaiacol (slightly sweet herbal incense), acetic acid Ding esters (strong fruit aroma), ethyl phenylacetate (similar to honey fragrant ester), phenethyl alcohol (sweet floral aroma), 3-methyl-pentanoic acid (sour herb smell, slightly green grass aroma), 2,6-dimethylpyrazine (roasted coffee, peanuts, potato aroma), furfural (sweet, roasted, woody), maltol (with butter, sugar, like a special focus fragrant aroma), lactic acid (mild cream aroma), benzaldehyde (bitter almond aroma), ethyl lactate (baked apple aroma), *n*-octanol (green fragrance, fruit, incense), 4-ethylphenol (phenolic wood aroma, slightly sweet aroma), 2,5-dimethyl-pyrazine (a strong focus scent), 2-acetylpyrrole (bread aroma) all had very high FD values (Log3 FD ≥ 4 ), which could account for the overall flavor of soypaste. Among them, the FD values of 2,3,5-trimethylpyrazine, isovalerate and 4-ethylguaiacol were as high as 2187 (Log3 FD = 7), which formed the bean flavor of the special paste aroma. According to the other similar studies [5], 2,3,5-trimethyl-pyrazine and 4-ethylguiacol were thought to be the characteristic compounds in the fermented bean aroma. It could be broadly inferred that the characteristic aroma of the soypaste should be attributed to the heterocyclic compounds and the organic acids. The role of heterocyclic compounds was particularly prominent, constituting the basis of the soy flavor. The results of this study should help us to control the fermentation process and improve the aroma of soypaste.

## 3. Experimental

### 3.1. Materials and chemicals

Soypaste was obtained from Fang Yuan Food Ltd. Company of Lan Yang City, Shang Dong Province. Other chemical were of analytical grade.

### 3.2. Fermentation process

After removing the impurities, the soybeans and wheat were cooked, cooled and mixed in a ratio of 6:4. The obtained mixture was used as the starter-material and fermented in a fermentation tank for six months. After the fermentation, it was collected as the soypaste.

### 3.3. SDE Preparation of aroma components from soypaste

The bean paste (200 g) was mixed with distilled water (300 mL) and a small amount of zeolite in a 1000 mL round-bottomed flask. The mixture was placed in a simultaneous distillation extraction instrument (Anhui Tianchang Excellent Letter Electrical Equipment Co., Ltd.) and extracted at 40 °C for 3 h. The extract was concentrated and dried by sodium sulfate for the subsequent GC-O and GC analysis.

### 3.4. Analysis of GC and GC-MS

GC-MS analysis was performed on an Agilent 6890NGC-5973IMS GC-MS. The gas chromatograph was equipped with a HP-INNO Wax Polyethylene Glycol capillary column 60 m × 0.25 mm coated with 0.25 μm film thickness. Carrier gas (helium) at flow rate of 1 mL/min. Column temperature program was 40 °C (3 min) isotherm, increased to 130 °C at a rate of 3 °C/min, maintained at 130 °C for 2 min, then increased to 200 °C at a rate of 4 °C/min and held at 200 °C for 5 min. The mass spectrometer was used in EI scan mode with a scan range of masses from 33 to 450 m/z. Ionization was set at 70 eV. Injector temperature was 250 °C. The compounds were identified by searching NIST02.1 database of the MSDChem workstation.

GC-O analysis was performed on an Agilent 7980 gas chromatograph interfaced to an ALPHA-MOS sniffer sniffing device (France ALPHA-MOS Corporation). The gas chromatograph was equipped with an Rtx-WAX capillary column 30 m × 0.25 mm coated with 0.25 μm film thickness (Agilent, USA). Carrier gas (helium) at flow rate of 1 mL/min. Column temperature program was 40 °C (3 min) isotherm, increased to 130 °C at a rate of 3 °C/min, maintained at 130 °C for 2 min, then increased to 200 °C at a rate of 4 °C/min and held at 200 °C for 5 min. The split ratio of the effluent into the FID and the ALPHA-MOS sniffer was 1:1. 

### 3.5. Analysis of AEDA *[6]*

The extract was diluted according to the volume ratios of 1:3, 1:9, 1:81, 1:243, respectively. The obtained sample (2 μL) was injected for the GC-O analysis until the evaluators at the GC-O terminal could not feel the smell. The obtained highest dilution ratio was defined as the FD factor. There were three professional evaluators from Central Research Institute of Ting Hsin International Group who performed the AEDA. 

## 4. Conclusions

In this study, the flavor substances of soypaste were extracted by simultaneous distillation method and subjected to standard analysis by GC-MS. The characteristic aroma components of soypaste were determined by the GC-O technique, and at the same time the FD values of the characteristic aroma components were determined by the AEDA method. The results could be used to evaluate the quality of soypaste.

## Figures and Tables

**Table 1:** Aldehyde and ketone components from the volatile compounds of soypaste.

	Name	RI		Smell description	Log3 FD
		WAX	DB-5		
1	2-Butyraldehyde	910	650	Nut odor	1
2	3-Butyraldehyde	914	664	Apple odor	3
3	Hexanal	1074	809	Aldehyde odor	<1
4	Heptaldehyde	1174	909	Fruit flavor, rose oil odor, perilla oil odor	3
5	5-*n*-Octyl aldehyde	1278	1014	Fruits, fat aroma and citrus odor	<1
6	Nonanal	1380	1102	Citrus and vinegar smell	2
7	3-Methylthiopropionaldehyde	1392	925	Potato and sauce smell	2
8	Furfural	1442	841	Sweet, roasted, woody, bread aroma	5
9	Benzaldehyde	1498	966	Bitter almonds, woody odor	4
10	5-Methylfurfural	1014	771	Thick, sweet, spicy odor,	2
11	Phenylacetaldehyde	1623	1059	Ocean narcissus elegant aroma, a strong wind letter sub-aroma, cherry flavor	2
12	2,3-Butanedione	973	606	Sweet cream, butter aroma	3
13	2,3-Pentanedione	1074	702	Sweet odor, cream odor.	2
14	3-Penten-2-one	1121	—	Fruit and spicy aroma	<1

**Table 2:** Alcohol or phenolic components from the volatile compounds of soypaste.

No	Name	RI		Smell description	Log3 FD
		WAX	DB-5		
1	Ethanol	930	—		2
2	iso-propyl carbinol	1080	—	Artificial musk aroma	<1
3	3-Methylbutanol	1205	739	Wines and ether smell	<1
4	Hexanol	1349	889	Fruit flavor	<1
5	1-Octen-3-ol	1443	978	Mushrooms, lavender, rose and hay aroma	2
6	Octanol	1550	1086	Citrus, sweet orange, aldehydes fragrant, sweet floral, fragrant aroma and green incense	4
7	Furfuryl alcohol	1660	—	Bitter and spicy smell	2
8	3-Methylthiopropanol	1436	1099	A strong aroma and taste of meat and broth, a strong smell of garden onions and meat, butter flavor when diluted	1
9	2-Methoxyphenol, guaiacol	1859	1102	Aromatic smell	<1
10	Phenylethanol	1885	1131	Sweet, floral aroma, and fruit, fat taste, rose aroma	6
11	Phenol	1920	—	Special smell, sweet smell	<1
12	4-Ethyl-2-methoxyphenol,	2032	—	Barbecue flavour	7
13	4 - Ethylphenol	2086	—	Wood phenol aroma, slightly sweet aroma	4
14	Maltol	1968	1108	butter, sugar, coke special aroma incense, strawberry flavor	5

**Table 3:** Ester components in the volatile compounds of soypaste.

No	Name	RI		Smell description	Log3 FD
		WAX	DB-5		
1	Ethyl acetate	885	<600	Ether fragrance, sweet fruit such as pineapple	3
2	Ethyl 2-methylbutyrate	1051	845	Strong apple skin, pineapple skin and immature sweet aroma of plum skin	<1
3	*n*-Butyl acetate	1070	820	Strong fruit aroma, similar to pear, banana aroma	6
4	Ethyl 3-methylbutyrate	1134	—	Similar to apple, bananas aromas and sweet and sour smell	<1
5	Ethyl caproate	1235	996	Fragrant fruits like pineapple and wine	4
6	Ethyl lactate	1293	825	Sweet, fruit, roasted, old rum aroma, wine aroma	
7	Octanoic acid ethyl ester	1431	1209	Brandy aroma, wax incense, milk and cream, fruit, wine	<1
8	Ethyl benzoate	1647	1188	Fruit, medicine fragrant aroma	1
9	Ethyl phenylacetate	1765	1258	Strong and sweet fragrance of honey. Significant and sweet incense rose	6
10	Ethyl palmitate	>2000	—	Incense wax smell, butter aroma	2

**Table 4:** Heterocyclic components in the volatile compounds of soypaste.

No	Name	RI		Smell description	Log3 FD
		WAX	DB-5		
1	2-Pentylfuran	1225	996	bean aroma, fruity, green fragrance, vegetables, fragrant soil, root incense aroma	2
2	2,5-Dimethylpyrazine	1316	925	Pungent aroma of fried flowers and chocolate, butter odor, roasted barley aroma, fried potatoes, fried potato chips	4
3	2,6-Dimethylpyrazine	1319	925	Roasted, coffee, peanuts, potato aroma and chocolate flavor	6
4	2,3,5-Trimethyl-pyrazine	1391	999	Baked goods, roasted barley, cocoa, coffee and pork, beef, popcorn, baked potatoes, roasted peanut odor	7
5	3-Ethyl-2, 5-dimethyl- pyrazine	1439	1071	Fried barley, cocoa products, coffee, peanut, hazelnut, soybean odor	3
6	2-Acetylfuran	1497	—	Almonds, nuts, fermented aroma, milk and sweet caramel-like aroma	1
7	1-Methoxy-4-(1- propenyl)-benzene, anethole	1682	1244	A special aroma of fennel	<1
8	2-Acetylpyrrole	1952	—	Bread aroma, bakery aroma	4

**Table 5:** Acidic components from the volatile compounds of soypaste.

No	Name	RI		Smell description	Log3 FD
		WAX	DB-5		
1	Acetic acid	1413	671	Acetic, vinegar odor	2
2	2-Methylpropionic acid	1468	—	Pungent odor, rancid oil odor	3
3	Butyric acid	1546	—	Milk, cream, butter, cheese, fruity aroma	2
4	3-Methylpentanoic acid	1597	882	Sour herbal aroma, slightly green grass aroma	6
5	Isovaleric acid	1638	966	Old sock odor	7
6	Hexanoic acid	1826	1209	Strong rancid cheese aroma	1
7	Lactic acid	>2000	1296	Mild and pleasant cheese aroma	5
8	Nonanoic acid	>2000	1308	Fats, wax, cheese, coconut fragrance	<1
